# Partial trisomy 21 with or without highly restricted Down syndrome critical region (HR-DSCR): report of two new cases and reanalysis of the genotype–phenotype association

**DOI:** 10.1186/s12920-022-01422-6

**Published:** 2022-12-21

**Authors:** Maria Chiara Pelleri, Chiara Locatelli, Teresa Mattina, Maria Clara Bonaglia, Francesca Piazza, Pamela Magini, Francesca Antonaros, Giuseppe Ramacieri, Beatrice Vione, Lorenza Vitale, Marco Seri, Pierluigi Strippoli, Guido Cocchi, Allison Piovesan, Maria Caracausi

**Affiliations:** 1 Department of Experimental, Diagnostic and Specialty Medicine (DIMES), Unit of Histology, Embryology and Applied Biology, University of Bologna, Via Belmeloro 8, 40126 Bologna, BO Italy; 2 Neonatology Unit, IRCCS Azienda Ospedaliero-Universitaria di Bologna St. Orsola Polyclinic, Via Massarenti 9, 40138 Bologna, BO Italy; 3 Medical Genetics Unit, University of Catania, Catania, Italy; 4 Cytogenetics Laboratory, Scientific Institute, IRCCS Eugenio Medea, Bosisio Parini, Lecco Italy; 5 U.O. Genetica Medica, IRCCS Azienda Ospedaliero-Universitaria di Bologna, Via Albertoni 15, Bologna, Italy; 6 Department of Medical and Surgical Sciences (DIMEC), University of Bologna, Via Massarenti 9, 40138 Bologna, BO Italy

**Keywords:** Down syndrome, Partial trisomy 21, Highly restricted Down syndrome critical region

## Abstract

**Background:**

Down syndrome (DS) is caused by the presence of an extra copy of full or partial human chromosome 21 (Hsa21). Partial (segmental) trisomy 21 (PT21) is the duplication of only a delimited region of Hsa21 and can be associated or not to DS: the study of PT21 cases is an invaluable model for addressing genotype–phenotype correlation in DS. Previous works reported systematic reanalyses of 132 subjects with PT21 and allowed the identification of a 34-kb highly restricted DS critical region (HR-DSCR) as the minimal region whose duplication is shared by all PT21 subjects diagnosed with DS.

**Methods:**

We report clinical data and cytogenetic analysis of two children with PT21, one with DS and the other without DS. Moreover, we performed a systematic bibliographic search for any new PT21 report.

**Results:**

Clinical and cytogenetic analyses of the two PT21 children have been reported: in Case 1 the duplication involves the whole long arm of Hsa21, except for the last 2.7 Mb, which are deleted as a consequence of an isodicentric 21: the HR-DSCR is within the duplicated regions and the child is diagnosed with DS. In Case 2 the duplication involves 7.1 Mb of distal 21q22, with a deletion of 2.1 Mb of proximal 20p, as a consequence of an unbalanced translocation: the HR-DSCR is not duplicated and the child presents with psychomotor development delay but no clinical signs of DS. Furthermore, two PT21 reports recently published (named Case 3 and 4) have been discussed: Case 3 has DS diagnosis, nearly full trisomy for Hsa21 and a monosomy for the 21q22.3 region. Case 4 is a baby without DS and a 0.56-Mb duplication of 21q22.3. Genotype–phenotype correlation confirmed the presence of three copies of the HR-DSCR in all DS subjects and two copies in all non-DS individuals.

**Conclusions:**

The results presented here are fully consistent with the hypothesis that the HR-DSCR is critically associated with DS diagnosis. No exception to this pathogenetic model was found. Further studies are needed to detect genetic determinants likely located in the HR-DSCR and possibly responsible for core DS features, in particular intellectual disability.

**Supplementary Information:**

The online version contains supplementary material available at 10.1186/s12920-022-01422-6.

## Background

Down syndrome (DS) is the most frequent chromosomal disorder, due to the presence of an extra copy of full or partial human chromosome 21 (Hsa21) [1]. DS affects approximately 1 in every 1000–1100 newborns around the world [2].

It is widely accepted that excess genetic material from Hsa21 is responsible for DS [3], but to date, there is not an exhaustive pathogenetic model that allows the linking of specific structural and functional elements of Hsa21 to the DS phenotype. Since the sequencing of Hsa21 in 2000, with the consequent mapping of Hsa21 genes [4], several genes have been candidated for DS-related phenotypes on the basis of product function, in particular 16 loci with a role in energy and reactive oxygen species metabolism, including *SOD1* (Superoxide dismutase 1, soluble); 9 loci affecting brain development, neuronal loss, and Alzheimer’s type neuropathology, including Single-minded homolog 2 (*SIM2* (Drosophila)), Dual-specificity tyrosine-(Y)-phosphorylation kinase 1A (*DIRK1A*) and Amyloid beta (A4) precursor (*APP*); and 6 loci with a role in folate and methyl group metabolism, including cystathionine beta-synthase (*CBS*) [5, 6]. Nevertheless, to date, we are not able to confirm the correlation of individual Hsa21 genetic elements to DS symptoms.

Partial (or segmental) trisomy 21 (PT21) reports describe genetic conditions with the duplication of only a delimited region of Hsa21. Several studies based on PT21 cases were performed over years to identify specific regions of Hsa21 responsible for DS characteristics: genotype–phenotype correlations have been obtained for several comorbidities associated with DS, in particular hypotonia [7], acute megakaryoblastic leukemia and transient myeloproliferative disorder [8, 9], congenital heart disease [10] or Alzheimer-like disease [11].

Instead of focusing on a specific sign or symptom, a different approach is considering the diagnosis of DS itself as the phenotype to be mapped: PT21 subjects can be classified as PT21 with DS diagnosis and PT21 without DS. In particular, subjects were classified as DS according to explicit statements found in the clinical reports, assessment of detailed phenotype description or when recognizable DS diagnosis is declared, independently of other characteristics associated to possibly concurrent chromosomal abnormalities. Subjects were classified as non-DS when a different distinct diagnosis has been described or individuals show normal phenotypes. Through this approach, the study of PT21 cases could provide a method to identify the “minimal” Hsa21 region associated to DS diagnosis and thus likely associated to the most typical DS characteristics such as a recognizable form of intellectual disability and some facial phenotypes. In fact, despite the presence of many differences in clinical and somatic characteristics of children with DS diagnosis, intellectual disability frequency is virtually 100% and typical facial phenotypes are the second most frequent signs of DS. Therefore, the genetic marker of these “core” features should be duplicated in all PT21 DS subjects and not duplicated in PT21 non-DS subjects, while variability of the phenotype may be affected by additive factors (allelic isoforms, copy number variants or CNVs, epistasis, positional effects in cases with translocations, epigenetics, environment). Our research group performed the most systematic search for any available cases published from 1973: a retrospective systematic reanalysis of 125 PT21 cases described up to 2015 allowed the creation of the most comprehensive PT21 map and the identification of the highly restricted DS critical region (HR-DSCR, 34 kbp in 21q22.13 Hsa21 region, from 37 929 229 to 37 963 130 [GRCh38]) as the minimal region whose duplication is shared by all PT21 subjects diagnosed with DS [12]. These data have been confirmed in a prospective study [13] updating the PT21 map (132 PT21 cases up to 2017) and supporting the “HR-DSCR model”: a critical region duplicated in all the PT21 subjects with DS but not duplicated in PT21 individuals without DS, thus likely associated to DS diagnosis and critical for “core” symptoms, in particular intellectual disability. No exception to this pathogenetic model was found. Due to the extreme rarity of PT21 cases, each new case can be informative and useful to validate the association between the HR-DSCR duplication and DS diagnosis.

The aim of this work is to report two new PT21 cases, the first with the duplication of the HR-DSCR and DS diagnosis, the second without the duplication of the HR-DSCR and signs and symptoms not suggestive of DS. Finally, we accurately searched in the biomedical literature for any new PT21 reports and we discuss recently published new cases.

## Methods

### Patients

The present study was approved by the independent Ethics Committee of St. Orsola-Malpighi Polyclinic, Bologna, Italy (approval no. 39/2013/U/Tess, multicentric study). Written informed consents were obtained from the parents of the children to collect clinical data and perform genetic studies. All methods were performed in accordance with the Ethical Principles for Medical Research Involving Human Subjects of the Helsinki Declaration.

#### Case 1

Case 1 was an Italian 2-year-old girl with PT21 and clinical diagnosis of DS. Patient enrollment was performed in the context of the routine follow up provided for DS.

#### Case 2

Case 2 was an Italian 9-year-old girl with PT21, without diagnosis of DS. Patient enrollment was performed in the context of a follow up for a psychomotor development delay.

### Clinical data

Clinical data were obtained during the routine follow up visits, including personal, genetic, diagnostic, clinical and auxological information from both the neonatal period and the time of the visit.

### Molecular cytogenetic characterization: FISH and array-CGH analysis

#### Case 1

Fluorescence in situ hybridization (FISH) analysis was carried out on samples of the proband and their parents on peripheral blood lymphocytes, according to standard techniques. FISH analysis was performed using Vysis Totelvysion DNA probes: D21Z1 (21p11.1-q11.1), wcp, AML, VIJyRM2029.

Array-based comparative genomic hybridization (array-CGH) analysis was performed using an Agilent SurePrint G3 ISCA v2 CGH 8 × 60 K microarray, with an average resolution of 120 kb (higher in ISCA regions), following the manufacturer's protocol (Agilent Technologies, Santa Clara, CA). Chromosomal imbalances were called through the ADM1 algorithm considering at least three consecutive oligonucleotides with similar log2ratio. A graphical visualization of the results was provided by the Genomic Workbench software v.7.0. In the present study genomic coordinates were converted to the matching current Genome Reference Consortium (GRC) human genome assembly GRCh38, or hg38, December 2013, using the online tool LiftOver (https://genome.ucsc.edu/cgi-bin/hgLiftOver).

#### Case 2

Array-CGH analysis was performed using an Agilent 180 K oligonucleotide array according to the manufacturer's protocol (Agilent Technologies, Santa Clara, CA). Data analysis was performed using Cytogenomics V.2.5.8.1.

To verify the presence and the parental origin of the unbalanced translocation der(20)t(20;21) suggested by microarray analysis, FISH analysis with subtelomeric probes D20S1157 (20p13) and D21S1146 (21q22.3) (Tel Vysion, Vysis Abbott Molecular, Des Plaines, Illinois, USA) was performed on metaphases from the proband and her parents. All the genomic coordinates related to previous versions of the human genome sequence were converted in the matching current coordinates on hg38 using the online tool LiftOver (https://genome.ucsc.edu/cgi-bin/hgLiftOver).

### Bibliographic searches and case selection from the literature

Following systematic bibliographic searches, 125 PT21 reports were identified from the literature and selected for the study of a critical region for DS [12]. Subsequently, the bibliographic search has been repeated and an updated analysis has been performed, building a map of a total of 132 PT21 cases with or without DS [13]. Here, we have repeated the bibliographic search to retrieve any new reports of PT21 and to integrate the new data in the previously published PT21 map [13]. In addition, weekly automated updates from NCBI reporting articles found with the “My NCBI” saved search: “Down Syndrome” [Mesh] OR “Down Syndrome” OR “Trisomy 21” were considered, to identify articles escaping the above search strategy.

We applied inclusion and exclusion criteria as previously described [12] in order to only include cases with sufficient and unambiguous description at cytogenetic, molecular and clinical levels. Briefly, the main cytogenetic inclusion criterion was the presence of duplication of a partial portion of 21q, in particular in the analysis we included trisomy 21 with one interstitial deletion or segmentally duplicated Hsa21; unbalanced reciprocal translocations involving segments of 21q; and tandem translocations with an incomplete long arm of the duplicated Hsa21. We excluded from the analysis cases presenting translocations and ring Hsa21 with a complete 21q; tetrasomies of Hsa21, a condition with a different gene dosage compared to trisomy 21; mosaic trisomy 21 because the cell mosaicism could affect the phenotype and confound the contribution of the partial duplication [14, 15]. In addition, we excluded chromosomal rearrangements involving Hsa21 and the X chromosome from the case selection because of the effects due to variable inactivation of Hsa21 regions translocated to X [16] and chromosomal alterations described in leukemic cell clones.

The molecular analysis criteria were a detailed and unambiguous description of the duplicated segment boundaries; availability of at least the banding pattern; availability of sequence data allowing placement of sequence tagged sites (STSs) and FISH probes on the map and coherence among different methods when they were used to study the same subject. All the genomic coordinates related to previous versions of the human genome sequence were converted in the matching current coordinates on hg38 using the online tool LiftOver (https://genome.ucsc.edu/cgi-bin/hgLiftOver).

At the clinical level, subjects were classified as DS or non-DS according to explicit statements found in the study; whether authors judged recognizable DS as present or absent, irrespectively of other symptoms or signs associated to possibly concurrent aneuploidies of non-Hsa21 chromosomal segments; or assessment of detailed phenotype description when present in the article. Fetuses were excluded due to the impossibility of ascertaining phenotype in detail [17].

## Results

### Patients

In this work we have reported the description of two patients with PT21: the first subject with a diagnosis of DS and the second one without diagnosis of DS but with a different type of psychomotor development delay.

Molecular data showed different trisomic regions, allowing the inclusion of these new PT21 cases in the study of the DS critical region.

### Clinical data

#### Case 1

The first proband is a 2-year-old Italian girl that is the first child of non-consanguineous healthy parents. Her parents were both 32 years old at the time of her birth and the mother did not have miscarriages before the pregnancy.

The diagnosis of DS was not established during pregnancy. During the first-trimester the nuchal translucency (NT) screening was normal (1 mm); the pregnancy-associated plasma protein A (PAPP-A) and free beta-human chorionic gonadotropin (bHCG) were 28,600 U/L (0.7198 MoM) and 2310 lU/L (0.6233 MoM) respectively: the risk of having a baby with DS was calculated as 1:497 (base-risk) and 1:7536 (correct-risk). The parents did not proceed with amniocentesis or chorionic villus sampling.

At 20 weeks of gestation, an ultrasound (US) showed the risk of complete atrioventricular septal defect (AVSD), later confirmed with the same exam at 23 + 5 weeks. Moreover, at 32 weeks of pregnancy another US scan revealed the presence of two adjacent fluid-filled echolucent structures within the abdomen of a fetus (commonly called “double bubble” sign) and polyhydramnios, leading to the suspect of duodenal atresia. As a consequence of the AVSD associated with duodenal atresia, our medical team suggested a possible diagnosis of DS.

The girl was born at 36 weeks of gestation, with natural delivery, as a consequence of premature rupture of the membranes. At the time of birth, the newborn’s APGAR score was 9 at 1 min and 9 at 5 min and her somatic features were: a weight of 2,550 g, and a recumbent length of 50 cm.

The analysis of dysmorphic features showed a clinical pattern compatible with diagnosis of DS (Table 1), also compatible with criteria suggested in the classic work by Jackson et al. [18] (Additional file 1: Table S1, 16/25 signs). Cytogenetic analysis was required to confirm the diagnosis.

**Table 1:** Main clinical features of Down syndrome observed in Case 1 and Case 2

Jackson's checklist	Case 1	Case 2	Frequency in DS subjects (%) [30]
Flat nasal bridge	+	**–**	86.7
Oblique eye fissure	+	**–**	85.1
Epicanthic eye fold	+	**–**	78.5
Excess of nuchal skin	–	**–**	60.3
Single transverse palmar crease (right, left)	+	**–**	60.3
Fifth finger mid-phalanx hypoplasia	+	**–**	51.2
Muscular hypotonia	+	N/A	40.4
Congenital heart defect	+ (AVSD)	**–**	24.7

+ : present; –: absent; N/A: data not available

Postnatal radiography showed a “double bubble” sign in the upper abdomen suggesting the presence of duodenal atresia or stenosis. No other gastrointestinal associated anomalies were detected, and the nasogastric output was clear and nonbilious. The child underwent a diamond-shaped-duodenoduodenostomy (DSD) on the second day of life, associated with complementary appendectomy. No complications related to the anastomosis were observed and postoperative oral feeding was progressively introduced from day IX after removal of the nasogastric tube.

The surgical repair of complete AVSD was performed at the VII month of life and the post-operative period was regular. Postoperatory pulmonary hypertension was treated with Sildenafil. The echocardiogram performed during the hospital stay showed normal cardiac dimension, normal cardiac contractility (65% fraction ejection of the LV) and a persistent IV defect, without a hemodynamic impact. At the cardiological evaluation at 1 year and 9 months of age, a balanced condition with a good health state and a normal growth trend of the child was observed. No residual signs of IV shunt at echocardiography test, while a mild mitral and tricuspid valve insufficiency was detected. No more signs of pulmonary hypertension and Sildenafil therapy was discontinued.

At 6 months of age an immunological evaluation was recommended due to low IgG value found in a routine blood analysis (IgG concentration = 95 mg/dL). The examination confirmed hypogammaglobulinemia, excluding alterations of lymphocyte subpopulations. Due to the low serum immunoglobulin levels, a first infusion of polyvalent immunoglobulins was scheduled in agreement with her parents; the therapy was followed by the reevaluation of basal immunoglobulin levels to monitor therapeutic efficacy.

The same year the child visited the Pediatric Emergency Department because of two episodes of abundant regurgitation: an x-ray exam of the digestive tract showed no gastrointestinal alterations. These signs suggested the diagnosis of esophageal reflux, later successfully treated with proton pump inhibitors therapy.

Regarding the auxological follow-up of the baby, the measurements: Length (L), Weight (W) and Head Circumference (HC) were performed at 3 months, then every 3 months during the first year and every 6 months during the second year of follow-up (Table 2). Referring to the growth velocity charts of the general population, the growth rate for the baby was below the 10th percentile, both in terms of L, W and HC. During the last year, due to reduction of comorbidities, an improvement in the growth rate reaching the 10-50^th^ percentile both for W and L, was observed. Nevertheless, using growth charts specific for children with DS, the pattern of L, W and HC were inside the normal percentile range, reaching almost the 90th for L and W, and the 50th of HC at 24 months (age at last evaluation).

**Table 2:** Auxological measurements performed during the follow-up of Case 1

	3 months	6 months	9 months	12 months	18 months	24 months
Recumbent length	56 cm	60 cm	66 cm	70 cm	77 cm	83
Weight	3.640 kg	4.820 kg	6.070 kg	8.080 kg	10.600 kg	11.300 kg
Head Circumference	35.5 cm	40 cm	41 cm	42.4 cm	44 cm	44.5 cm

#### Case 2

The second proband is a 9-year-old Italian girl who was born at 39 (2/7) weeks of gestational age by spontaneous vaginal delivery as the second pregnancy of healthy and non-consanguineous parents. Her mother and father were respectively 38 and 29 years old when she was born.

The first child was a boy affected with X- linked agammaglobulinemia, the mother being a carrier. The second pregnancy was spontaneous and villocentesis, performed only for detection of fetal sex and considered negative, was followed by abortion threats. There was mild intrauterine growth retardation. Prenatal screening was not performed. Prenatal ultrasound examinations were normal. The mother did not smoke and denied any alcohol intake during pregnancy.

Regarding anthropometry at birth, her head circumference was 31.5 cm, length was 48.5 and weight was 2810 g. The Apgar score test, performed one minute after birth, was 9/10. The child demonstrated normal adaptation to extrauterine life, joint laxity of the hips and congenital clubfoot corrected with physiotherapy.

The child walked autonomously around 18 months and spoke first words after 12 months, but she never acquired age-appropriate language. She had no convulsion, but the mother noticed she had reduced memory abilities.

**Fig. 1:**
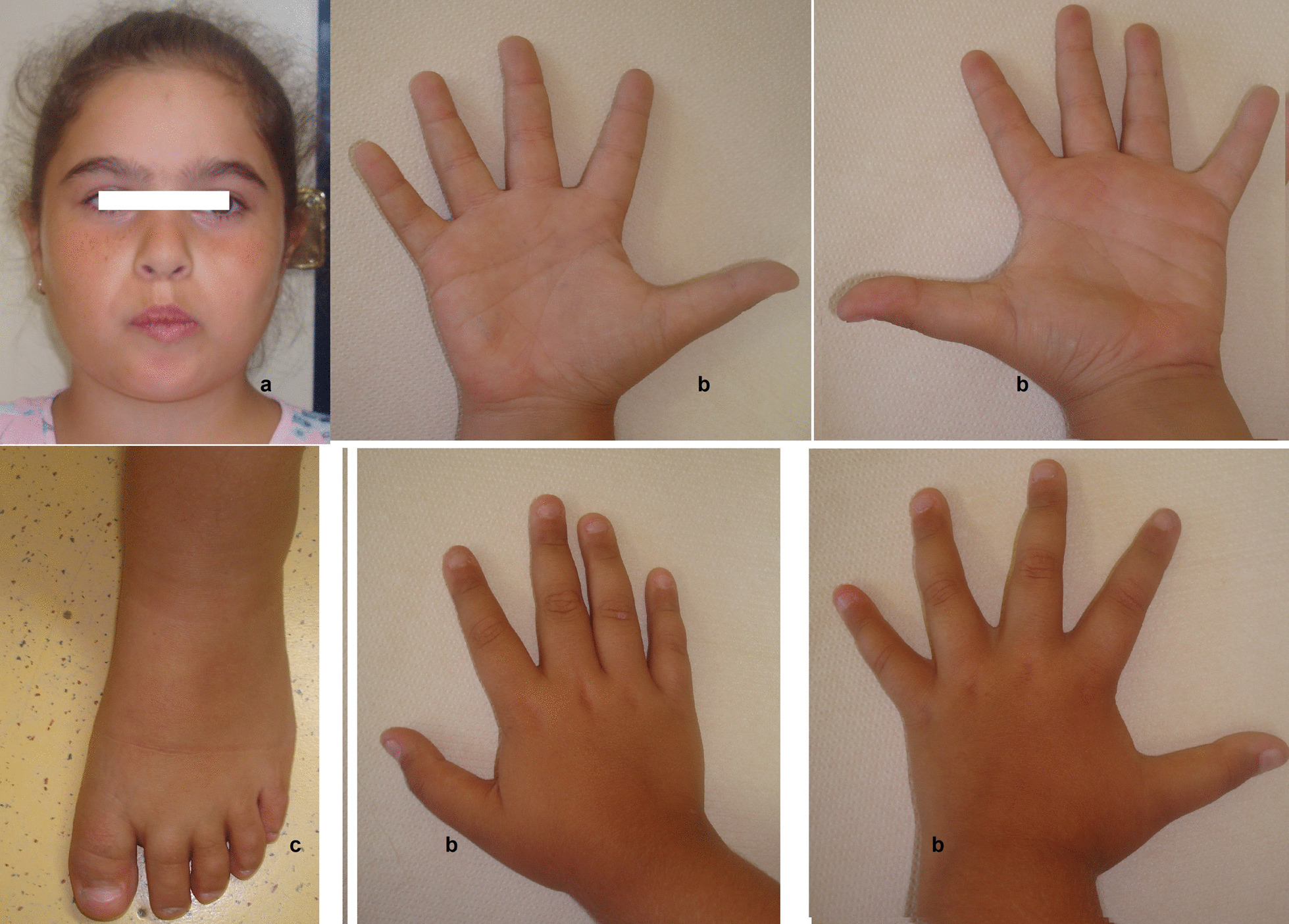
Picture of Case 2 showing her face (**a**), hands (**b**) and left foot (**c**). She does not show the Down syndrome recognizable phenotype

At 9 years of age (age at last evaluation), she was referred to the Medical Genetics Unit, because of psychomotor development delay and then, intellectual disability. At physical examination, she did not show the DS recognizable phenotype (Fig. 1), and peculiar dysmorphic features could not be detected on her face or body (Table 1 and Additional file 1: Table S1, < 5 signs). Therefore, according with the clinical team, we have classified the girl as non-DS. Attention-Deficit/Hyperactivity Disorder (ADHD) was diagnosed, along with a sleep disorder. The patient is also overweight, has hypertrichosis and shows early pubarca.

### Molecular cytogenetic characterization: FISH and array-CGH analysis

#### Case 1

**Fig. 2:**
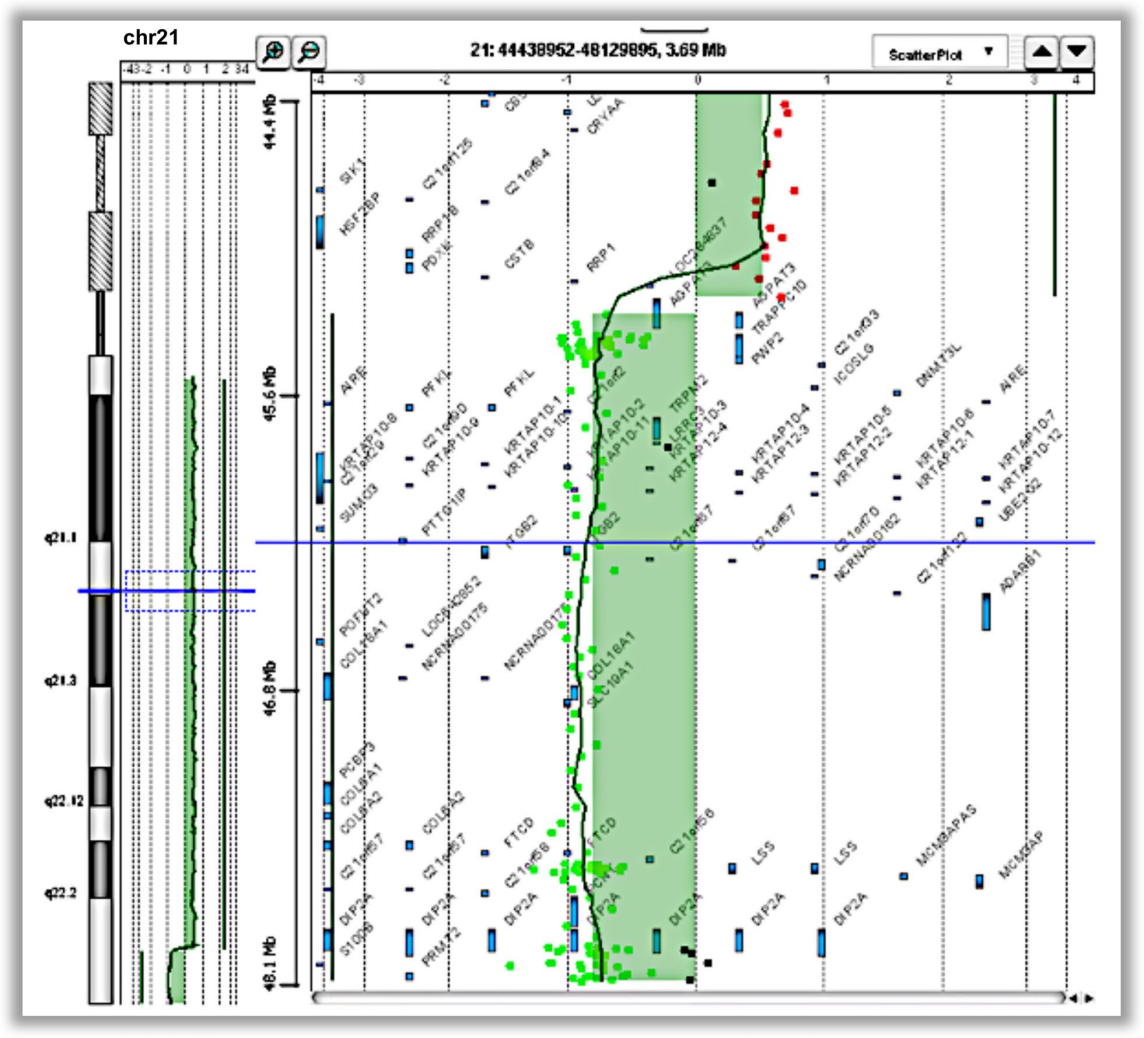
Array-CGH analysis of DNA from Case 1 showing the alteration of chromosome 21 (chr21): arr[GRCh38] 21q11.2q22.3(14145727_43860444) × 3,21q22.3(43927315_46670405) × 1. In the present study genomic coordinates were converted to the matching current coordinates on hg38 using the online tool LiftOver (https://genome.ucsc.edu/cgi-bin/hgLiftOver) so the figure shows the duplication of chromosome 21 from 14,145,727 to 43,860,444 bp and the deletion from 43,927,315 to 46,670,405 bp (GRCh38). The HR-DSCR (chr21 from 37,929,229 to 37,963,130) is within the duplicated regions

The metaphasic FISH analysis of DNA from Case 1 showed a homogeneous trisomy deriving from an isodicentric chromosome 21, with breakpoints in the 21q22.3 chromosomal region. This analysis also revealed the loss of the terminal part of the long arm of both chromosomes forming the isodicentric one. According to ISCN 2020 nomenclature, the alterations may be described as: 46,XX,idic(21)(q22.3)dn.ish idic(21)(D21Z1++,WCP+,AML++,VIJyRM2029–). FISH analysis was performed on the parents, showing two normal Hsa21 in both. Moreover, array-CGH analysis of the proband showed a duplication from 14,145,727 to 43,860,444 bp (29.715 Mb) and a deletion of about 2.7 Mb from 43,927,315 to 46,670,405 bp (GRCh38), with the following ISCN 2020 nomenclature arr[GRCh38] 21q11.2q22.3(14145727_43860444) × 3,21q22.3(43927315_46670405) × 1 (Fig. 2).

#### Case 2

**Fig. 3:**
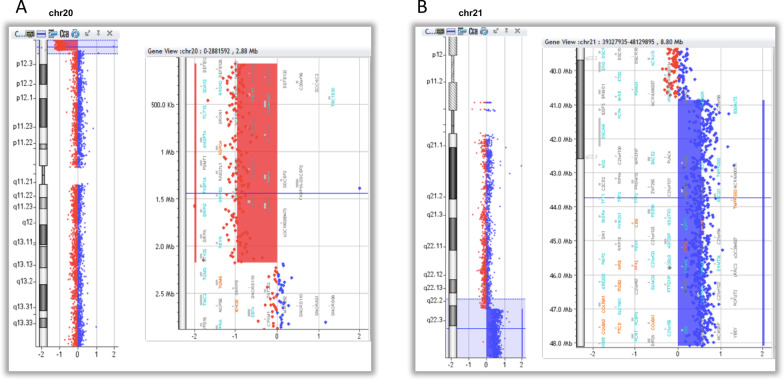
Array-CGH analysis of DNA from Case 2 showing the alteration of chromosome 20 and 21: [GRCh38] 20p13(87137_2197493) × 1, 21q22.2q22.3(39502312_46670405) × 3. In the present study genomic coordinates were converted to the matching current coordinates on hg38 using the online tool LiftOver (https://genome.ucsc.edu/cgi-bin/hgLiftOver). **A** Array-CGH analysis showing the deletion of chromosome 20 (chr20) from 87,137 to 2,197,493 bp (GRCh38). **B** Array-CGH analysis the duplication of chromosome 21 (chr21) from 14,145,727 to 43,860,444 bp and the deletion from 43,927,315 to 46,670,405 bp (GRCh38). The HR-DSCR (chr21 from 37,929,229 to 37,963,130) is not duplicated

Array-CGH analysis revealed a 20p13 distal deletion of 2.1 Mb and a 21q22.2q22.3 distal duplication of 7.2 Mb (Fig. 3), suggesting the presence of an unbalanced translocation der(20)t(20;21).

FISH analysis on metaphases confirmed that the proband carried an unbalanced translocation, as the subtelomere probe for 20p (D20S1157) was present in the normal chromosome 20 and absent in the derivative chromosome 20 on which the subtelomere probe for 21q (D21S1146) was transposed.

FISH analysis on parental metaphases showed that father harbors the balanced translocation.

Thus the final interpretation of the rearrangement, accordingly with the ISCN 2020, was: ish der(20)t(20;21)(p13−;q22.3+)(D20S1157−;D21S1146+)dpat.arr[GRCh38] 20p13(87137_2197493) × 1, 21q22.2q22.3(39502312_46670405) × 3.

### Bibliographic searches and case selection from the literature

Bibliographic searches resulted in 92 new papers. Only two studies reported PT21 cases matching the inclusion and exclusion criteria described in the Methods section named Case 3 [19] and Case 4 [20] in the present work.

#### Case description

Case 3 [19] is a female baby with DS. The karyotype with an isodicentric chromosome resulting in a partial trisomy 21 was 46,XX,idic(21)(q22.3). A chromosomal microarray analysis (CMA) confirmed the presence of nearly full trisomy for chromosome 21 and also a monosomy for the 21q22.3 region; in particular the chromosomal alteration has been described as arr[GRCh38]21p11.2q22.3(10810857_45448165) × 3,21q22.3(45471378_46664244) × 1.

Case 4 [20] is a male baby without DS phenotype except congenital heart disease (CHD). Molecular analysis revealed a 0.56-Mb duplication of 21q22.3 described as arr[GRCh38] 21q22.3(46062296_46623792) × 3.

## Discussion

Complete or partial trisomy 21 is the genetic cause of DS. A useful model for linking genotype and phenotype in DS is the study of the very rare genetic condition called PT21, the duplication of only a delimited segment of Hsa21. Systematic attempts were published in 2009 to identify “critical regions” on Hsa21 for several distinct phenotypes observed in DS [7, 8].

Our approach to phenotype-genotype correlation was focused on the diagnosis of DS itself as the phenotype to be mapped, rather than trying to identify subregions responsible for distinct phenotypes [12, 13]. Especially in the cases of borderline phenotypes and complex chromosomal rearrangements, physical examination is the most accurate initial diagnostic assessment, and an experienced clinician will recognize the physiognomic features, often accompanied by muscular hypotonia, that may suggest or not the diagnosis of DS [21]. Moreover, collection and analysis of the 25 physical signs recognized in the classic work by Jackson et al. [18] as the most discriminating signs for DS diagnosis, can be a useful tool that can help clinicians in evaluation. As recently demonstrated [22], Jackson’s checklist has a current validity in clinical use: individuals with 13 or more signs or with less than 5 signs can correctly be diagnosed as affected or non-affected. In the present work, subjects were therefore classified as DS or non-DS according to the clinical team evaluation supported by the analysis of Jackson’s checklist.

Systematic analyses of PT21 cases could provide a method to identify the “minimal” Hsa21 region associated to diagnosis of DS, thus likely associated to DS “core” features, in particular intellectual disability.

**Fig. 4:**
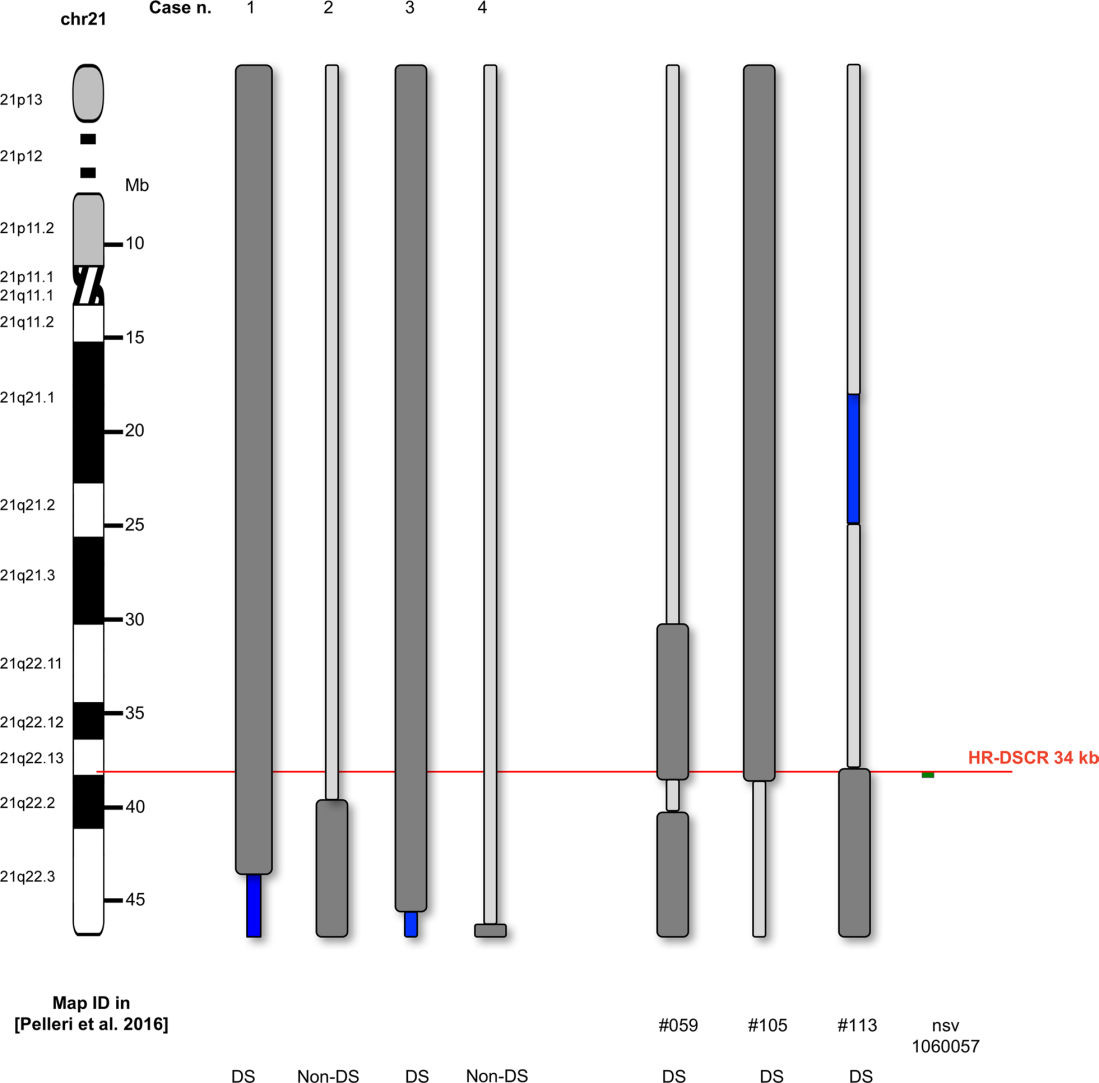
HR-DSCR as highlighted by the partial trisomy 21 integrated map (simplified view). The cases described in the present work are shown (Cases 1, 2, 3, 4); moreover, the cases (#059, #105 and #113, intellectual disability used in [12] and the copy number variant (CNV, nsv1060057, from Database of Genomic Variants, http://dgv.tcag.ca/) strictly defining HR-DSCR limits are shown here. Light grey bar: disomic region; dark grey bar: trisomic region: Blue bar: monosomic region. Case n. 1 (this work); Case n. 2 (this work); Case n. 3 [19]; Case n. 4 [20]; #059: Case DUP21SOL [8]; #105: [29]; #113: Case DUP21HAD [8]. DS: subject with Down syndrome; non-DS: subject without Down syndrome

In the present work, we described detailed clinical reports and cytogenetic characterizations of two probands with PT21 (Cases 1 and 2, Table 3 and Fig. 4), but different phenotypes. In case 1, a 2-year-old girl, the duplication involves the whole long arm of Hsa21, except for the last 2.7 Mb, which are deleted as a consequence of an isodicentric 21: the HR-DSCR is within the duplicated regions and the child is diagnosed with DS. A variable clinical picture is reported in patients with pure 21q22.3 deletions, both in literature [23] and in public clinical databases, such as ClinVar and DECIPHER. An accurate phenotypic comparison between patients with pure deletions and Case 1 is complicated by scarce clinical details reported in databases and by larger sizes of deletions published in literature. We cannot exclude that the 2.7 Mb deletion at 21q22.3 and potentially additional low fraction cell lines due to the mitotic instability of isodicentric chromosome 21 and not detectable in blood might contribute to Case 1 phenotype. However, isodicentric chromosome 21 has been previously reported in patients with a consistent DS phenotype, with slight variations likely due to the deleted terminal region [19].

**Table 3:** Summarized data about PT21 cases mapped in this work

Case	Diagnosis	Sex	Age	Karyotype	Coordinates (GRCH38)	Method	Country	Reference
1	DS	F	0–2 yrs	46,XX,idic (21)(q22.3)	21q11.2q22.3 (14,145,727-43,860,444) × 3, 21q22.3 (43,927,315-46,670,405) × 1	FISH aCGH	Italy	This work
2	Non-DS	F	0–9 yrs	46,XX,Der(20)t(20;21) (p13-;q22.3+)	20p13 (87,137 2,197,493 × 1, 2,210,152 × 2), 21q22.2q22.3 (39,486,350 × 2, 39,502,312-46,670,405 × 3)	FISH aCGH	Italy	This work
3	DS	F	1 mos	46,XX,idic(21)(p11.2->q22.3::q22.3->p11.2)	21p11.2q22.3 (10,810,857-45,448,165) × 3 21q22.3 (45,471,378-46,664,244) × 1	Banding FISH aCGH	Pennsylvania (US)	[19]
4	Non-DS	M	6 mos	46,XY	21q22.3 (46,062,296-46,623,792)3	aCGH	Taiwan	[20]

Cases 1 and 2 are first described here and cases 3 and 4 are retrieved from the biomedical literature

M, male; F, female; FISH, fluorescence in situ hybridization; aCGH, array-CGH

In case 2, a 9-year-old female, the duplication involves 7.1 Mb of distal 21q22, with a deletion of 2.1 Mb of proximal 20p, as a consequence of an unbalanced translocation: the HR-DSCR is not duplicated, and the child presents with psychomotor development delay but the clinical condition not classifiable as DS. The 2.1 Mb deletion on chromosome 20p13 likely has a major role in the pathogenesis of the observed phenotype. Indeed, 20p13 deletions have been reported in patients with motor and speech developmental delays, intellectual disability, epilepsy and non-specific dysmorphic features [24]. Moreover, the deletion includes the *CSNK2A1* gene, associated to the autosomal dominant Okur-Chung neurodevelopmental syndrome (MIM 617062), mainly characterized by global developmental delay, intellectual disability, autism spectrum disorder, attention deficit hyperactivity disorder, motor disorder, and many behavioral disorders, including sleep problems [25]. Although missense mutations have been predominantly reported, they ultimately lead to a reduction of the kinase activity of the encoded protein, in a haploinsufficient or hypomorphic manner, suggesting a functional effect similar to truncating mutations or deletions [26].

However, for the purpose of this study it remains clear that diagnosis of DS is associated to the presence in three copies of the HR-DSCR (Case 1), while two copies of the region have not been sufficient to lead to diagnosis of DS in Case 2.

Then, we accurately searched for any new PT21 reports published in the last year in order to update the integrated comparative map and possibly confirm the HR-DSCR model.

Our bibliographic searches identified two clinical cases of interest (Cases 3 and 4, Table 3 and Fig. 4). Case 3 is a female newborn with DS that, despite the presence of unusual defects like esophageal atresia and tethered cord syndrome, has a clear DS phenotype [19]. Indeed, the karyotype showed the trisomy of approximately the whole long arm of the Hsa21 excluding a segment of almost 1.2 Mb at the 21q22.3 terminal, which turned out to be monosomic. Case 4 is a male baby without DS and a familial 21q22.3 microduplication [20].

Finally, it would be worthwhile to discuss a further report from the literature [27]: a 5 1/2-year-old boy with a clear clinical diagnosis of DS and an interesting cytogenetic profile. Indeed, FISH and array-CGH analysis showed a mosaic pattern with a microduplication in approximately 40% of lymphocytes and in approximately 80% of buccal mucosa cells, involving 2.56 Mb of the chromosomal region 21q22.13q22.2, in particular arr[GRCh38]21q22.13q22.2(37296053_39815527) × 3 dn. In previous works, we decided to exclude all cases with mosaic trisomy 21 from the analysis because of the effects of cell mosaicism on the subject’s phenotype. In fact, this cytogenetic condition could confound the proband’s phenotype, hindering DS diagnosis and making medical reports difficult to interpret. Therefore, this PT21 case cannot be included in the analysis but it deserves a discussion. Despite the mosaic pattern, the subject has a clear DS phenotype and, furthermore, he carries the smallest duplications within the DSCR leading to a DS clinical pattern ever described. In fact, this case alone could be sufficient to exclude almost 94.6% of Hsa21 as associated to the diagnosis of DS. Remarkably, in this subject a full copy of the HR-DSCR is included in the duplicated region.

These findings underline the need for a better undesrtanding of the structure of the HR-DSCR, which has been poorly investigated. Very recently, we have challenged the concept that this is an intergenic region, as it appears from the human genome browsers. By both in silico and in vitro analyses, *KCNJ6-202* and *DSCR4-202* isoforms have been identified [28]. *KCNJ6-202* shares the coding sequence with the known transcript *KCNJ6-201*, encoding a potassium channel which is involved in many physiological processes, including heart rate in cardiac cells and circuit activity in neuronal cells. *DSCR4-202* transcript has the first two exons in common with *DSCR4-201*, the only experimentally verified gene uniquely present in Hominidae. Further research is needed to assess any role of these HR-DSCR transcripts, or other transcripts to be identified, in the DS phenotype.

## Conclusions

In conclusion, PT21 cases are a very useful model for the identification of critical regions associated to specific phenotypes. Our efforts were oriented to a better understanding of the phenotype-genotype correlation in DS and, in this regard, we focused on DS diagnosis itself as the phenotype to be mapped. Using this approach, we wanted to evaluate whether the HR-DSCR model (34 kb on Hsa21 likely to be critical for DS diagnosis), first described by Pelleri and Coll. [12, 13], could prospectively be confirmed.

Through the study of selected literature cases and the description of two new PT21 clinical cases, we were able to update the PT21 map previously published [13]: in total, 137 PT21 cases, 96 of which with DS and 41 without DS. Our results are fully consistent with the concept that the HR-DSCR is critical for DS diagnosis being the only duplicated sequence shared by all DS subjects. However, both reviewed and new cases did not allow us to refine the HR-DSCR limits because the breakpoints of their trisomic segments turned out to be outside the HR-DSCR.

The study of each PT21 case can be crucial to studying the HR-DSCR model as to date no exception has been demonstrated.

## Supplementary Information

**Additional file 1: Table S1**. A complete list of the 25 Jackson's physical signs reported for Case 1 and Case 2 and their frequencies reported in Jackson et al. [18].

## Data Availability

All data generated or analysed during this study are included in this published article.

## Abbreviations

DS: Down syndrome
Hsa21: Chromosome 21
PT21: Partial trisomy 21
HR-DSCR: Highly restricted DS critical region
NT: Nuchal translucency
PAPP-A: Pregnancy-associated plasma protein A
bHCG: Free beta-human chorionic gonadotropin
US: Ultrasound
AVSD: Atrioventricular septal defect
PROM: Premature rupture of the membranes
DSD: Diamond-shaped-duodenoduodenostomy
L: Length
W: Weight
HC: Head Circumference
ADHD: Attention-Deficit/Hyperactivity Disorder
CMA: Chromosomal microarray analysis
CHD: Congenital heart disease
FISH: Fluorescence in situ hybridization
array-CGH: Array-based comparative genomic hybridization
GRC: Genome Reference Consortium
STSs: Sequence tagged sites
CNV: Copy number variant
