# Supplementary material for: Partial trisomy 21 with or without highly restricted Down syndrome critical region (HR-DSCR): report of two new cases and reanalysis of the genotype–phenotype association
Source: BMC Med Genomics. 2022 Dec 21;15:266. doi: 10.1186/s12920-022-01422-6 (PMC9768891; doi:10.1186/s12920-022-01422-6)
Supplement: Supplementary file 1 — Additional file 1: Table S1. A complete list of the 25 Jackson's physical signs reported for Case 1 and Case 2 and their frequencies reported in Jackson et al. [18]. [file 12920_2022_1422_MOESM1_ESM.docx]

**Additional file 1: Table S1.** A complete list of the 25 Jackson's physical signs reported for Case 1 and Case 2 and their frequencies reported in Jackson et al. [18]

| Jackson's Checklist | Case 1 | Case 2 | Frequency in DS subjects (%) [28] |
| --- | --- | --- | --- |
| Flat nasal bridge | + | **-** | 86.7 |
| Oblique eye fissure | + | **-** | 85.1 |
| Epicanthic eye fold | + | **-** | 78.5 |
| Brachycephaly | + | **-** | 75.2 |
| Short neck | - | **-** | 70.2 |
| High-arched palate | + | **-** | 67.7 |
| Narrow palate | + | **-** | 67.7 |
| Separated hallux | + | **+** | 64.4 |
| Short and broad hands | - | **-** | 61 |
| Excess of nuchal skin | - | **-** | 60.3 |
| Single transverse palmar crease (right, left) | + | **-** | 60.3 |
| Joint laxity | + | - | 59.5 |
| Fifth finger mid-phalanx hypoplasia | + | **-** | 51.2 |
| Folded ear/helix | - | **-** | 42.9 |
| Incurved fifth finger (right, left) | + | **-** | 42.9 |
| Mouth permanently open | - | **-** | 40.4 |
| Muscular hypotonia | + | N/A | 40.4 |
| Protruding tongue | - | **-** | 38.0 |
| Brushfield spots (iris color) | N/A | - | 34.7 |
| Heart murmur | **+** | **-** | 33.0 |
| Abnormal teeth | - | **-** | 31.4 |
| Congenital heart defect | **+ (**AVSD) | **-** | 24.7 |
| Blepharitis, conjunctivitis | + | **-** | 22.3 |
| Furrowed (plicated) tongue | - | **-** | 22.3 |
| Nystagmus | + | - | 17.3 |
